# Exploring diazoxide and continuous glucose monitoring as treatment for Glut1 deficiency syndrome

**DOI:** 10.1002/acn3.51462

**Published:** 2021-10-06

**Authors:** Santhi N. Logel, Ellen L. Connor, David A. Hsu, Rachel J. Fenske, Neil J. Paloian, Darryl C. De Vivo

**Affiliations:** ^1^ Division of Pediatric Endocrinology and Diabetes University of Wisconsin‐Madison School of Medicine and Public Health Madison Wisconsin USA; ^2^ Department of Neurology University of Wisconsin‐Madison School of Medicine and Public Health Madison Wisconsin USA; ^3^ Clinical Nutrition University of Wisconsin Hospital and Clinics Madison Wisconsin USA; ^4^ Division of Pediatric Nephrology University of Wisconsin‐Madison School of Medicine and Public Health Madison Wisconsin USA; ^5^ Departments of Neurology and Pediatrics Columbia University Irving Medical Center New York New York USA

## Abstract

Glut1 deficiency syndrome is caused by *SLC2A1* mutations on chromosome 1p34.2 that impairs glucose transport across the blood–brain barrier resulting in hypoglycorrhachia and decreased fuel for brain metabolism. Neuroglycopenia causes a drug‐resistant metabolic epilepsy due to energy deficiency. Standard treatment for Glut1 deficiency syndrome is the ketogenic diet that decreases the demand for brain glucose by supplying ketones as alternative fuel. Treatment options are limited if patients fail the ketogenic diet. We present a case of successful diazoxide use with continuous glucose monitoring in a patient with Glut1 deficiency syndrome who did not respond to the ketogenic diet.

## Introduction

Glut1 deficiency syndrome (Glut1DS) is caused by mutations in *SLC2A1* on chromosome 1p34.2, which impairs transmembrane glucose transport across the blood–brain barrier resulting in hypoglycorrhachia and decreased glucose availability for brain metabolism.^1^ The consequence is a drug‐resistant metabolic epilepsy due to energy deficiency.^2^ Standard treatment for Glut1DS is the ketogenic diet (KD) but treatment options are limited if patients fail the KD.^3^ Diazoxide, which inhibits insulin release, was used sparingly in the past (De Vivo, unpublished observations) for five Glut1DS patients to increase blood glucose levels and thus intracerebral glucose levels. Unfortunately, their treatment was complicated by unacceptable persistent hyperglycemia with blood glucoses in the 300–500 mg/dL range. However, chronic hyperglycemia as treatment for Glut1DS remains sensible as it has been shown that acute hyperglycemia produces transient neurological improvement in Glut1DS patients.^4^

Continuous glucose monitoring (CGM) was first approved by the FDA in 1999. However, it was not widely used in pediatrics until 2015 after significant technological advances were made. CGM is commonly used by individuals with diabetes mellitus and studies have shown reductions in hemoglobin A1c and hypoglycemia with consistent use.^5^, ^6^, ^7^, ^8^, ^9^, ^10^, ^11^, ^12^, ^13^, ^14^, ^15^, ^16^ CGM works through a small sensor inserted subcutaneously. The sensor measures interstitial glucose levels every 5–20 min. Interstitial glucose measurements generally correlate well with blood glucose levels, although interstitial values can lag behind blood levels if the latter are changing rapidly. CGM is also being investigated for use in congenital hyperinsulinism, and early studies show benefit in trending glucose levels and guiding the need for supplemental meter checks.^17^, ^18^ Given these potential benefits, we employed the use of CGM to enable initiation and titration of diazoxide in a patient with KD‐resistant Glut1DS.

## Case Report

### Diazoxide initiation using CGM

**Figure 1:**
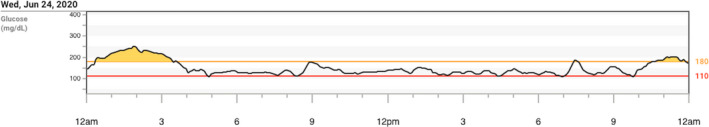
First day with home continuous glucose monitor.

A 14‐year‐old girl with Glut1DS (c.398_399delGCinsTT:p. Lys133Phe) presented with absence seizures at age 2 years. At initial presentation, she had a developmental and epileptic encephalopathy dominated by continual absence seizures and mild cognitive impairment. She also had dysarthria, hyperreflexia, and wobbly legs as a measure of paroxysmal exertional dyskinesia, often precipitated by athletic activities. Laboratory tests revealed a CSF glucose of 36 mg/dL when blood glucose was 93 mg/dL (CSF/blood glucose ratio 0.39). A classic KD was initiated with a 4:1 ratio and transitioned to a modified Atkins diet with a carbohydrate allowance as high as 50 g/day. Nevertheless, she could not tolerate the KD due to severe nausea, vomiting, abdominal pain, and hypertriglyceridemia. Trials of levetiracetam, zonisamide, valproic acid, carbamazepine, lacosamide, alpha lipoic acid, and triheptanoin also failed to control seizures. At age 14 years, diazoxide was started with target blood glucoses of 120–180 mg/dL. She was on no other anti‐seizure medication at that point. Serial EEG seizure counts documented a seizure‐free state after 6 months of diazoxide (Table 1). Seizures were identified as generalized 3‐Hz spike‐and‐slow wave discharges >5 s; pre‐diazoxide studies had indicated such discharges were clinically correlated with impaired responsiveness. CGM was placed at month 2 of diazoxide and showed an average interstitial glucose of 157 mg/dL with glucose variability of 20.8% (goal <36%) while on diazoxide 7.3 mg/kg/day. This diazoxide dose was within the typical range of 5–20 mg/kg/day used for the treatment of hypoglycemia due to congenital hyperinsulinemia. At home, CGM was used to adjust diazoxide doses 2–4 times a week to achieve target interstitial glucoses of 140–180 mg/dL (Fig. 1). Repeat laboratory tests at month 8 of diazoxide revealed a CSF glucose of 55 mg/dL when the blood glucose was 118 mg/dL (CSF/blood glucose ratio 0.47). Current diazoxide dose is 7.6 mg/kg/day, targeting interstitial glucoses of 90–110 mg/dL (glucose variability 17.1%). Most recent hemoglobin A1c was 5.7%.

**Table 1:** Glucose, diazoxide, and EEG data of the patient with Glut1 deficiency syndrome at time points relative to initiation of diazoxide therapy. Based on electroclinical correlation, seizures were identified as generalized 3‐Hz spike‐and‐slow wave discharges longer than 5 sec.

Time point	Glucose levels	Diazoxide dose	EEG seizure count
−2 months	–	–	84 per 24 h (2 day study)
3 weeks	92–145 mg/dL (blood)	9.0 mg/kg/day	6 per 24 h (3 day study)
2 months	124–190 mg/dL (interstitial)	7.3 mg/kg/day	4 per 24 h (3 day study)
6 months	140–284 mg/dL (interstitial)	7.9 mg/kg/day	0 per 24 h (5 day study)
8 months	80–201 mg/dL (interstitial)	8.4 mg/kg/day	0 per 24 h (1 day study)

**Figure 2:**
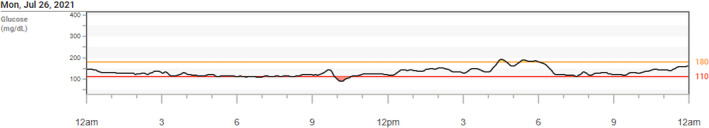
Recent day with home continuous glucose monitor.

Prior to diazoxide, the patient performed 3 grade levels below chronological age. At month 5 of diazoxide, at the start of grade 9, she performed at a 5.8 grade level in reading comprehension. By month 12 of diazoxide, reading comprehension improved to the 6.9 grade level. She continues to be seizure‐free and EEG tracings remain normal. She routinely reports her day is a 10 on a scale of 1 to 10. She is now more physically active and participates in Special Olympics basketball, softball, swimming, golf, and track (Fig. 2).

### Adverse events

#### Management of diazoxide‐induced fluid retention

The patient’s clinical course has been complicated by fluid retention, a known side effect of diazoxide.^19^ She developed this complication soon after diazoxide initiation. Diazoxide causes fluid retention by increasing sodium reabsorption at the distal tubule of the kidney.^20^ This mechanism was addressed using thiazide diuretics, which inhibit sodium reabsorption at the distal tubule. The patient began daily hydrochlorothiazide 1 month after diazoxide initiation.

At month 8 of diazoxide, the patient developed headache, malaise, and a stiff neck. She did not have papilledema. Lumbar puncture showed opening pressure of 22 cm H_2_O with benign cell counts. Because there was immediate relief of headache after the lumbar puncture, she was started on acetazolamide empirically for suspected low‐pressure intracranial hypertension. At month 9 of diazoxide, she developed hypokalemia (lowest potassium 2.8 mmol/L) and renal insufficiency (peak creatinine 1.11 mg/dL) necessitating transient discontinuation of hydrochlorothiazide. Hydrochlorothiazide was restarted later at a low dose with amiloride (potassium‐sparing diuretic). Daily sodium intake was decreased to <2400 mg/day. Serum electrolytes and renal function were followed closely. Acetazolamide was discontinued after normalization of serum sodium and renal function.

#### Weight gain and nutritional therapy on maintenance diazoxide

To achieve hyperglycemia in the range of 140–180 mg/dL, cornstarch was supplemented at 7 tbsp/day. Simple carbohydrates were given, when necessary, to raise blood glucose levels. Weight gain (13.7 kg) resulted from this regimen, complicating the fluid retention. Nutrition assessment revealed an average intake of 3193 kcal/day composed of carbohydrates (426 g/day), protein (121 g/day), and fat (108 g/day), which exceeded daily requirements of ˜2000 kcal/day. Her nutrition plan was restructured. The family was counseled regarding daily dietary intake to meet the patient’s energy, sodium, and carbohydrate goals. Daily cornstarch dose was decreased and eventually discontinued. To prevent relative “hypoglycemia”, the patient and family were educated about pairing complex carbohydrates with protein at meals and snacks to help sustain blood glucose levels. If relative “hypoglycemia” did occur, defined as blood glucose <90 mg/dL for more than 3–4 h, the patient and family were instructed to correct with 15 g of simple carbohydrates. Three weeks later, average daily caloric intake had decreased over 400 kcal/day. The patient’s nutritional status continues to be monitored closely as her neurological state continues to improve.

## Discussion

This is the first report demonstrating CGM as a tool facilitating safe initiation and real‐time titration of diazoxide in the management of Glut1DS patients who have failed the KD. Diazoxide addresses neuroglycopenia physiologically by raising blood glucose levels. The reasons and benefits for this are twofold. First, the Glut1 transporter facilitates glucose diffusion across tissue barriers including the blood–brain barrier. Reported Km values for the transporter range from 4 to 8 mmol/L (72–144 mg/dL).^21^, ^22^, ^23^ By increasing resting blood glucose values from 75 to 150 mg/dL, one can anticipate increasing brain glucose levels by an equivalent amount and attenuating the fuel mismatch that exists between brain glucose supply and demand in Glut1DS. It is critical to address this mismatch early in life when neurological growth and development are rapid as the cerebral metabolic rate for glucose reaches its peak between ages 3 and 8 years. Second, ketones enter the citric acid cycle at the acetyl‐CoA pool in the mitochondria. Pathways upstream of this pool that feed into or are fed by the glycolytic pathway, such as glycogen synthesis, pentose phosphate pathway, and lactate‐pyruvate metabolism, are not benefitted by the KD. This highlights glucose as the natural, preferred fuel for brain metabolism with ketones serving as a supplemental fuel source.

We observed previously that the higher blood glucose levels we achieved with chronic diazoxide administration only correlated with transient benefit (unpublished data). Ultimately, the Glut1DS patients lost the initial clinical benefits and regressed to baseline levels of neurological performance. We speculated chronic hyperglycemia (300–500 mg/dL) caused downregulation of *SLC2A1* adding “insult to injury”. This molecular sequence has been documented in poorly controlled diabetes mellitus.^24^, ^25^, ^26^ CGM use has allowed us to avoid such complications as witnessed by the success in this case study. Thus, CGM introduces the possibility of diazoxide becoming an alternative standard of care for Glut1DS and also may provide a valuable tool for long‐term management of other disorders of carbohydrate metabolism.

A multidisciplinary team approach has been central to the success of this patient’s care. The specialized team includes neurologists, endocrinologists, genetic counselor, nephrologist, and nutritionist. Diazoxide initiation was coordinated by neurologists and endocrinologists well versed in Glut1DS and diazoxide, respectively. Side effects caused by diazoxide were discussed among the endocrinologists managing the diazoxide, the neurologists following the developmental/epileptic encephalopathy and headache symptoms, a nephrologist managing the diuretics, and the nutritionist managing the dietary therapy. Team meetings were conducted regularly to assess treatment efficacy and re‐evaluate treatment goals. Fortunately, the parents and patient were able to assume increasing responsibility for the CGM and dietary adjustments. The outcome has been beneficial and resulted in optimal control of neurological symptoms and unprecedented quality of life.

## Conflict of Interest

The authors declare no conflict of interest. Darryl C De Vivo has served as advisor/consultant for AveXis, Biogen, Cytokinetics, Ionis Pharmaceuticals, Inc., Metafora, Roche, Sanofi, Sarepta, and SMA Foundation; received grants from Hope for Children Research Foundation, National Institutes of Health, SMA Foundation, Cure SMA, Glut1 Deficiency Foundation and US Department of Defense; clinical trial funding from Biogen, Mallinckrodt, PTC, Sarepta, Scholar Rock, and Ultragenyx; and as a member of the DSMB for aspa Therapeutics.
